# Avenanthramide C Suppresses Matrix Metalloproteinase-9 Expression and Migration Through the MAPK/NF- κB Signaling Pathway in TNF-α-Activated HASMC Cells

**DOI:** 10.3389/fphar.2021.621854

**Published:** 2021-03-25

**Authors:** Junyoung Park, Hyunju Choi, Fukushi Abekura, Hak‐Seong Lim, Jong‐Hwan Im, Woong‐Suk Yang, Cher‐Won Hwang, Young‐Chae Chang, Young-Choon Lee, Nam Gyu Park, Cheorl-Ho Kim

**Affiliations:** ^1^ Department of Biological Sciences, College of Science, Sungkyunkwan University, Suwon, South Korea; ^2^ Department of Biotechnology, College of Fisheries Sciences, Pukyong National University, Busan, South Korea; ^3^ Nodaji Co., Ltd., Pohang, Republic of Korea; ^4^ Department of AGEE, Handong Global University, Pohang, South Korea; ^5^ Research Institute of Biomedical Engineering and Department of Medicine, College of Medicine, Catholic University of Daegu, Daegu, South Korea; ^6^ Faculty of Medicinal Biotechnology, Dong-A University, Busan, South Korea

**Keywords:** anti-atherosclerosis, MMP, TNF-α, HASMCs (human aortic smooth muscle cells), avenanthramide C

## Abstract

In oat ingredients, flavonoids and phenolic acids are known to be the most important phenolic compounds. In phenolic compounds, wide-ranging biological responses, including antioxidative, anti-inflammatory, anti-allergic, and anti-cancer properties, were reported. Avenanthramide C (Avn C), a component of the phenolic compound of oats, has been reported to be highly antioxidant and anti-inflammatory, but its role in an anti-atherosclerosis response is unknown. The aim of this research was to assess the effect of Avn C on expression of MMP-9 on TNF-α-activated human arterial smooth-muscle cells (HASMC) and signaling involved in its anti-atherosclerosis activity. HASMC cells are known to produce inflammatory cytokines involving IL-6, IL-1β, and TNF-α during arteriosclerosis activity. Avn C specifically reduced IL-6 secretion in HASMC cells. Furthermore, we investigated whether Avn C could inhibit NF-κB nuclear protein translocation. Avn C suppressed nuclear protein translocation of NF-κB in TNF-α-stimulated HASMCs. The MMP-9 enzyme activity and expression are controlled through the MAPKs signaling path during the Avn C treatment. We confirmed that the levels of wound healing (*p*-value = 0.013, *p < 0.05) and migration (*p*-value = 0.007, **p < 0.01) are inhibited by 100 ng/ml TNF-α and 100 μM Avn C co-treated. Accordingly, Avn C inhibited the expression of MMP-9 and cell migration through the MAPK/NF-κB signaling pathway in TNF-α-activated HASMC. Therefore, Avn C can be identified and serve as disease prevention material and remedy for atherosclerosis.

## Introduction

Matrix metallopeptidases (MMP) play an important role in angiogenesis, cell behavior, differentiation, cell proliferation, migration, and host defense (Kuzuya and Iguchi, 2003; Flamant et al., 2007). MMP-9 can disassemble the extracellular matrix (ECM) and is prominent in the vulnerable areas of atherosclerosis (Watanabe and Ikeda, 2004; Johnson, 2017). MMP-9 and pro-inflammatory cytokines are significant factors that enable the disassembly of the ECM. Also, MMP is well known as the major cause of atherosclerosis (Beaudeux et al., 2004; Flamant et al., 2007). Atherosclerosis is a cause of vascular disease. Thus, the importance of research on metastasis and treatment, a disease of arteriosclerosis, is gradually increasing. MMP-9 and MMP-2 play a significant role in the migration of HASMC cells into the inner membrane of blood vessels, which further exacerbates many vascular diseases and atherosclerosis (Visse and Nagase, 2003; Wagsater et al., 2011; Park et al., 2019).

**FIGURE 1:**
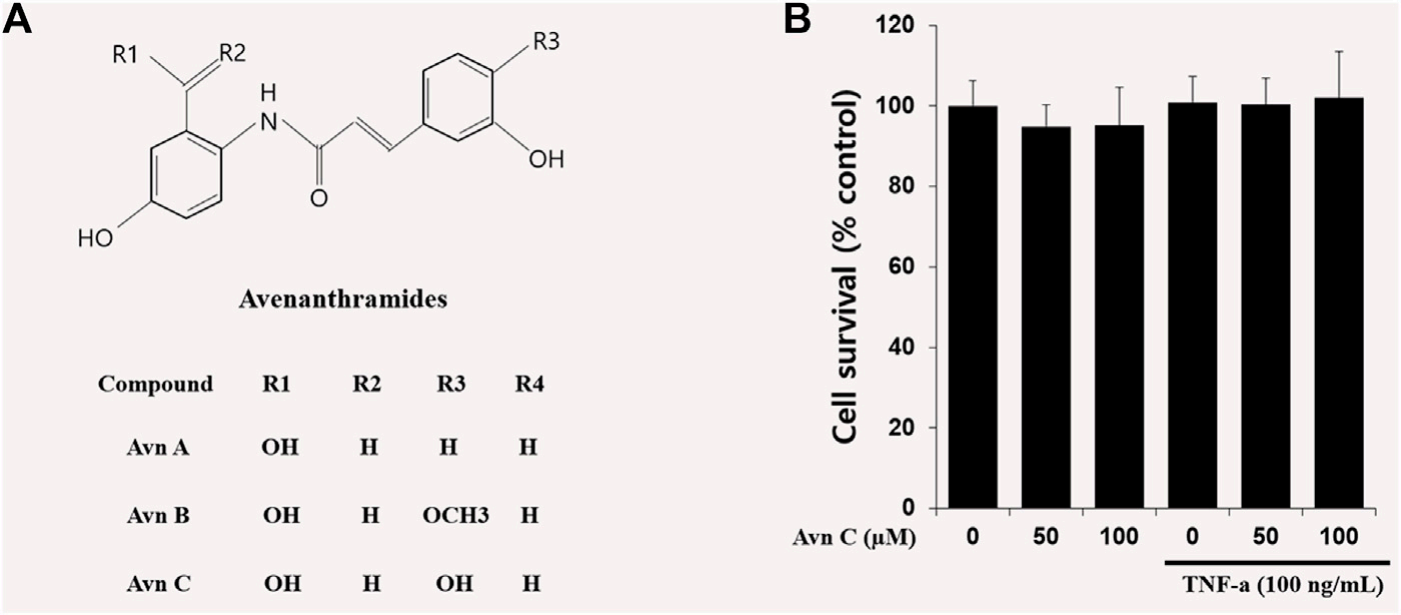
Chemical structures of Avenanthramides and effects on cell viability in HASMC cells.**(A)** Avenanthramides: AvnA, AvnB and AvnC (Chemical structures) **(B)** Cell viability measured by MTT. 5 × 10^3^ cell/well cells were treated with Avn C (0, 50, or 100 µM) and TNF-α (100 ng/ml) for 24 h.

In oat ingredients, phenolic acid and flavonoid are the most important phenolic compounds. The phenolic compounds exhibit wide-ranging biological responses as antioxidants, anti-inflammatory, anti-allergic and anti-cancer agents (Cebeci and Sahin-Yesilcubuk, 2014; Meydani, 2009; Singh et al., 2013). Avenanthramides (Avns) are combinations of a 5-hydroxy anthranilic acid and phenylpropanoid. Avns extracted from oats are soluble phenolic compounds (Bei et al., 2020; Ishihara et al., 2014). Three main isoforms of Avn, A, B, and C (Figure 1A) have been verified, and the previous results have shown their anti-histamine, anti-tumor proliferative, anti-oxidant, and anti-inflammatory functions (Sur et al., 2008).

Recently, there have been several reports that Avn C restores impaired plasticity and cognition in an Alzheimer's disease model with mice and suppresses eccentric exercise-inflicted blood inflammatory markers (Koenig et al., 2016; Ramasamy et al., 2020). It has also been reported that Avn C decreases the cell survival of MDA-MB-231 cells via an apoptotic system and protects noise and drug-activated hearing loss by protecting auditory hair cells from oxidative stress (Hastings and Kenealey, 2017; Umugire et al., 2019). But the role of Avn C in anti-atherosclerosis responses is unknown.

In this study, we used TNF-α-induced HASMC cells as an arteriosclerosis model. The results showed that the Avn C decreased MMP-9 expression of HASMC cells activated with TNF-α dose-dependently. In addition, we studied the effect of Avn C on anti-arteriosclerosis activity with TNF-α treatment to find out its molecular mechanisms.

## Materials and Methods

### Reagents

Avenanthramide C was purchased from Sigma–Aldrich (St. Louis, MO, United States; cat. no. 36465) and dissolved in dimethyl sulfoxide (DMSO) (St. Louis, MO, United States; cat.no. D2650) used in doses of 1 μM. It has a purity of 98% by SDS-PAGE gel and HPLC analyses and the grade is analytic standard. Human recombinant TNF-α protein (Purity: 98%) was purchased from PeproTech (Rocky Hill, NJ, United States). Griess reagent (cat. no. G4410), Hoechst staining solution (cat. no. 94403), 3- (4,5-dimethylthiazol-2-yl)-2,5-diphenyltetrazolium bromide (MTT) (cat. no. M5655), Corning trans-well Wright-Giemsa stain solution and 6-diamidino-2-phenylindole dihydrochloride (DAPI) (cat. no. D9542) were purchased from Sigma–Aldrich (St. Louis, MO, United States). p-ERK, ERK, p-p38, p-JNK, JNK and NF-kB were purchased from Cell signaling technology (Danvers, MA, United States). And, MMP-9, MMP-2, p38, β-actin, p-IkB and IkB antibodies were purchased from Santa Cruz Biotechnology (Paso Robles, CA, United States).

### Cell Culture and MTT Assay

Human Aortic Smooth. Muscle Cells (HASMC) were purchased from American Type Culture Collection (ATCC; Rockville, MD, USA). These cells were cultured in DMEM (WelGENE Co., Daegu, Korea) after processing by 10% metal bovine serum (FBS), 100 U/ml penicillin, and 100 mg/ml streptomycin (P/S) on each medium in a humidified atmosphere containing 37°C and 5% CO_2_ incubator. To assess cell viability, we seeded HASMC cells into a 96-well dish at a density of 1 X 10^4^ cells/well and treated them with Avn C (0, 50, or 100 µM). We added MTT solution and incubated the cells for 4 h at 37°C in a CO2 incubator. We solubilized the reaction reagent with dimethyl sulfoxide (DMSO) and measured optical density at 550 nm after 15 min of incubation.

### Gelatin Zymography Assay

Cultural medium with 100 ng/ml TNF-α with or without 100 μM Avn C treatment was collected from 12-well plates. The sample was mixed with a 5% Tris-glycine SDS sample buffer and loaded into 10% polyacrylamide gel with 1 mg/ml gelatin. After being run at 110 V for 1 h 20 min, the gel was washed with 2.5% Triton X-100 for 20 min and then incubated with incubation buffer (10 mM Tris base, 40 mM Tris–HCl, 200 mM NaCl, and 10 mM CaCl2) at 37°C for 12 h. The gel was then dyed for 2 h with comassie blue.

### Western Blot

We extracted the protein from the HASMC cells using the 1% NP40 protein lysis buffer containing 100 mM Na-Northovanadate, 100 mM NaF, and 100 mM Na-pyrophosphate and centrifuged the extract at 4°C at 13,000 rpm. The extracted protein was quantified by Bio-Rad protein assay (Bio-Rad Laboratories Hercules, CA, United States) and separated the same amount (by size) of protein through SDS-PAGE (SDS-polyacrylamide). Then, we transferred the membrane to the NC (nitrocellulose) membrane, reacted it to the primary antibodies [MMP-9 (cat.no. sc-13520, 1:2000), MMP-2 (cat. no.13594, 1:2000), β-actin (cat. no. sc-47778; 1:3000), NF-κB (cat. no. sc-372; 1:1000), Lamin B (cat no. sc-365962,1:1000), p-IκB (cat no. sc-8404,1:2000), IκB (cat. no. sc-1643, 1:2000), p-ERK (cat. no. 9101S; 1:2000), ERK (cat. no. 9101S; 1:2000), p-JNK (cat. no. #9255S; 1:2000), JNK (cat. no. 9252S; 1:2000), p-p38 (cat. no. sc-7975; 1:2000) and p38 (cat. no. sc-535; 1:2000) from Santa Cruz Biotechnology and Cell signaling technology, and horseradish peroxidase-linked anti-mouse (#31430, 1:2000), anti-rabbit (#31460, 1:2000), or anti-goat (#31402, 1:2000) immunoglobulin G secondary antibodies from Invitrogen and Thermo], and checked the results through the ECL (Enhanced chemiluminesence).

### Reverse Transcription-Polymerase Chain Reaction (RT-PCR)

Total RNA of HASMC cells was extracted using TRizol agent (Invitrogen, USA), and the cDNA was synthesized using the oligo dT primer using RT-Premix kit (Takara Shuzo, Japan). The cDNA was amplified by a PCR machine using these primers: MMP-9, forward 5′-TCC​CTG​GAG​ACC​TGA​GAA​CC-3′, reverse 5′-CGG​CAA​GTC​TTC​CGA​GTA​GTT-3′; MMP-2, forward 5′-TGA AGG TCG GTG TGA ACG GA-3′ and reverse 5′-CAT GTA GCC ATG AGG TCC ACC AC-3′; and β-actin, forward 5′-TCC​TTC​TGC​ATC​CTG​TCG​GC-3′, reverse 5′-CAA​GAG​ATG​GCC​ACG​GCT​GC-3′.

### ELISA

We measured the levels of inflammatory cytokines such as TNF-α, IL-1β and IL-6 in culture medium using ELISA kits (Affymetrix, eBioscience) along with antibodies for each cytokine according to the protocol.

### Immunofluorescence Assay

TNF-α was treated on HASMC cells, which were fixed 24 h later using 4% PFA (paraformaldehyde), increased permeability to 0.2% Triton-100, sequentially reacted with a primary antibody, a secondary antibody, and DAPAI, and checked by fluorescence microscope.

### Trans-Well Migration Assay

We added the HASMC cells to the top of the transwell, added the TNF-α-treated badges to the bottom, and incubated them for 24 h. The cells moved through the 8-mm pore-size membrane, were fixed through methanol, were dyed with Giamsa, and then the dyed cells were identified by means of an optical microscope.

### Wound Healing Assay

HASMCs were seeded into a 6-well dish at a density of 3 × 10^5^ cells/well. The wound was artificially scratched with a yellow pipette tip (200 μl) and washed to a moderate extent three times. After 24 h of processing either Avn C or Avn C + TNF-α, each well was observed microscopically.

### Statistical Analysis

All experiment results are representative of at least three independent experiments. The results of the data were assessed by one-way analysis of variance (ANOVA), followed by a post hoc Bonferoni test; a *p* < 0.05 was considered statistically significant. *p < 0.05 and **p < 0.01 indicate significant differences from the TNF-α-alone-treated cells.

## Results

### Chemical Structures of Avenanthramides and Effects on Cell Viability in HASMC Cells

The chemical structures of Avn A, B and C (Figure 1A) of the major phenolic components of oats are specifically features of 5-hydroxy anthranilic acid and phenylpropanoid. The molecular weight of Avn C is 315.28 g/mol. To study the Avn C-influenced cell viability in HASMC cells, we did an MTT assay to assess the cellular proliferation. We could not observe any cell death following treatment with Avn C (0–100 μM) in the absence or presence of 100 ng/ml TNF-α. (Figure 1B). Thus, we have used 0–100 μM Avn C for all of the experiments.

### Avn C Suppresses TNF-α-Stimulated Migration and Wound Healing of HASMC Cells

**FIGURE 2:**
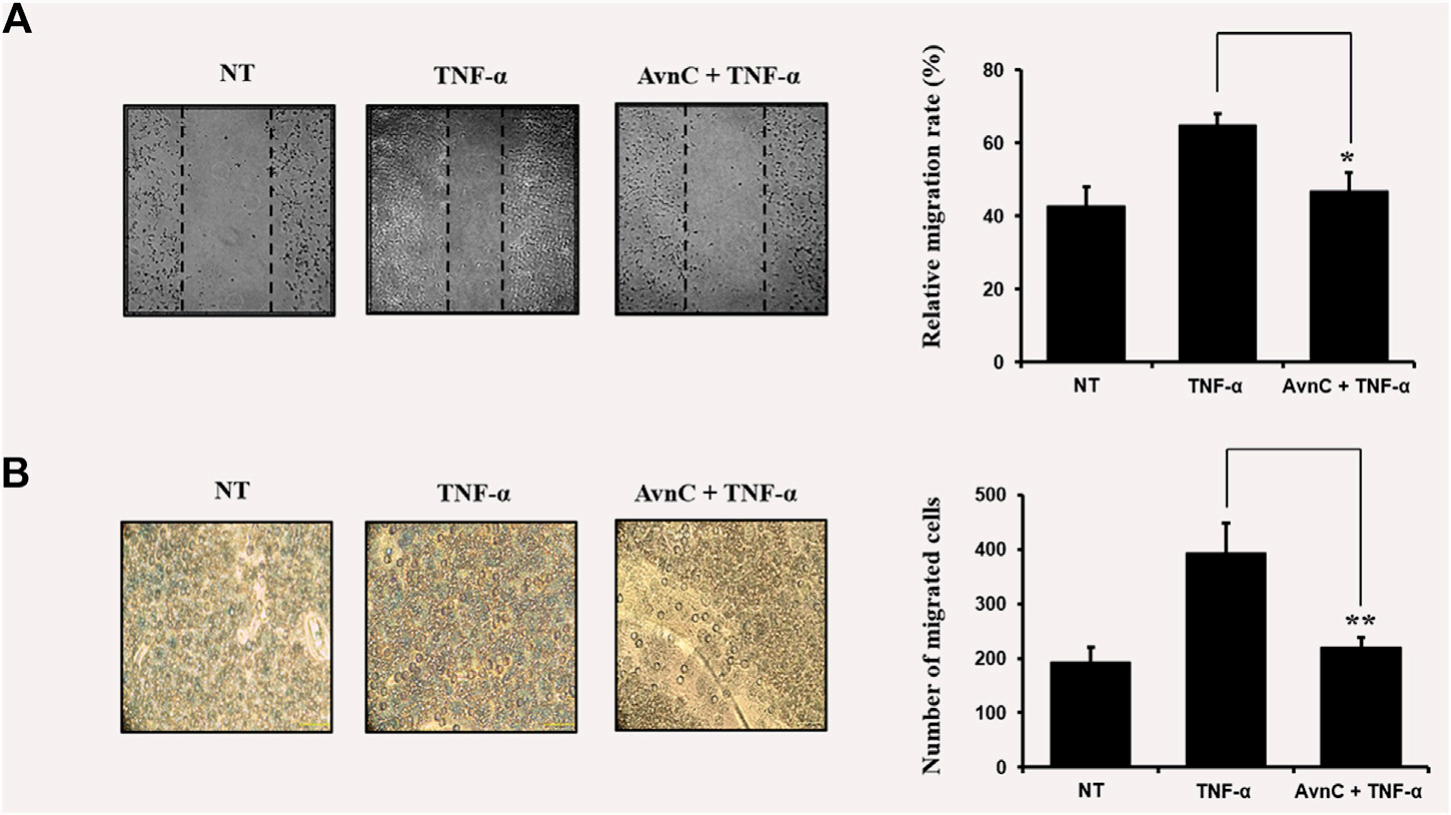
Avn C inhibits TNF-α -stimulated migration and wound healing of HASMC cells **(A)** HASMC cells were scratched with a yellow pipette tip. The cells were treated with 100 ng/ml TNF-α with or without 100 μM Avn C for 24 h. Cells migrated into the wound area **(B)** The migration ability of HASMC cells was detected by a transwell assay. The cells were incubated with 100 ng/ml TNF-α with or without 100 μM Avn C for 24 h. Results shown are representative of three independent experiments. They are presented as mean ± SEM. *p < 0.05 and **p < 0.01, significant differences from TNF-α treated cells. NT, no treatment.

TNF-α has been reported to be involved in metastasis of tumor cells or invasive vascular cells (Balkwill, 2006). To confirm the effect of Avn C on the cell migration and wound healing in TNF-α-activated HASMC cells, the following experiments were conducted. As shown in Figures 2A,B, when TNF-α is treated with HASMC cells, wound healing and migration levels are increased. But when TNF-α and Avn C are co-treated, the levels of wound healing and migration are inhibited.

### Effects of Avn C on Levels of mRNA and Protein Expression as Well as Enzyme Activities of mmp-9 and mmp-2 in HASMC Cells

**FIGURE 3:**
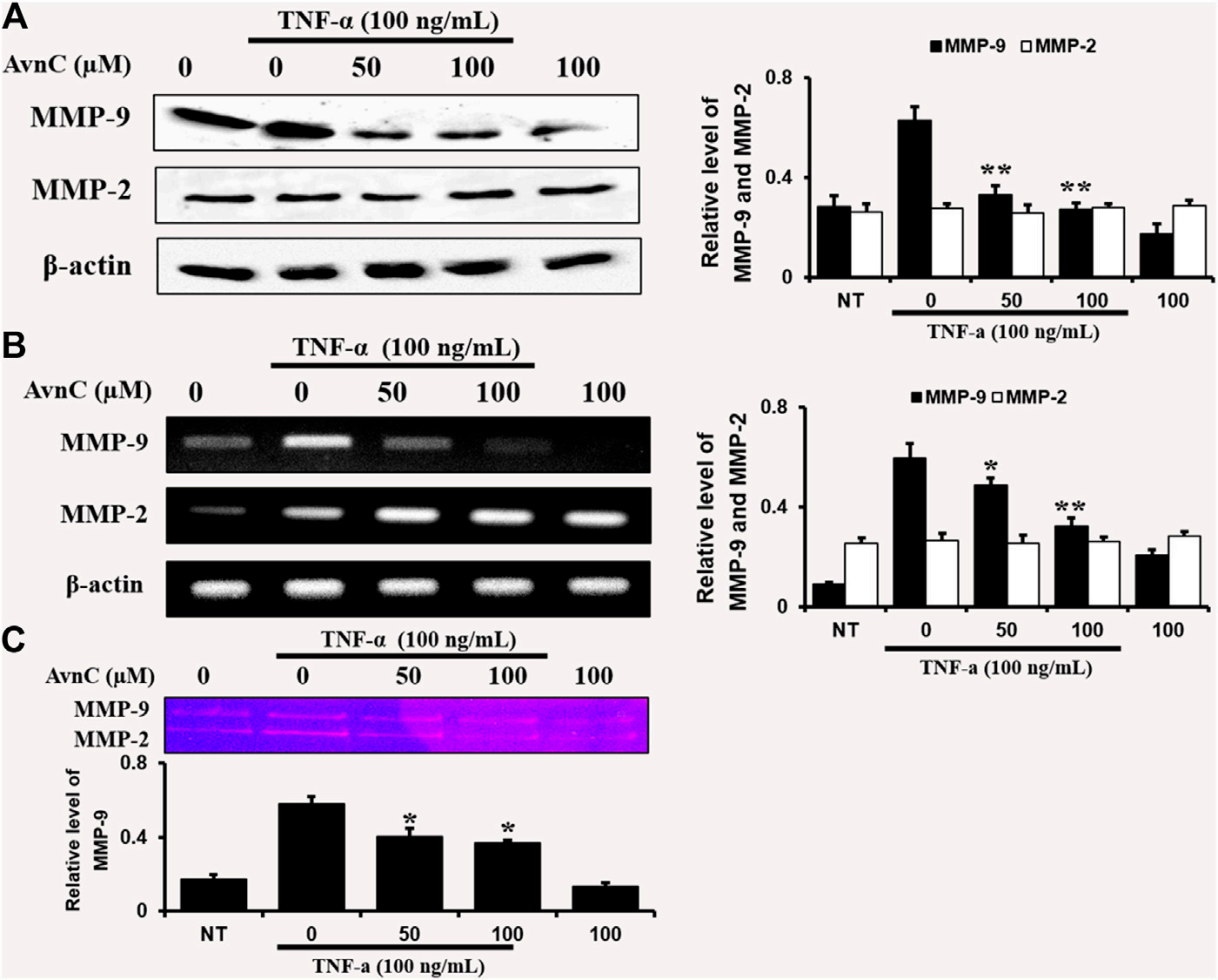
Effects of Avn C on TNF-α-induced MMP-9 and MMP-2 expression levels in HASMC cells.HASMC cells were co-treated with 0–100 μM Avn C and 100 ng/ml TNF-α for 24 h. Protein **(A)** and mRNA **(B)** levels respectively were examined by Western blotting and RT-PCR **(C)** Gelatin zymography results of MMP-9 enzyme activity levels. Results shown are representative of three independent experiments. They are presented as mean ± SEM. *p < 0.05 and **p < 0.01, significant differences from the TNF-α treated cells. NT, no treatment.

To find out the anti-atherosclerotic effect of Avn C, we assessed the levels of protein expression of MMP-9 and MMP-2 in the cells stimulated by TNF-α (100 ng/ml) by Western blot, RT-PCR, and gelatin zymography analysis. The levels of protein and mRNA expression were evaluated after incubating HASMC cells with 100 ng/ml TNF-α and 0–100 μM Avn C for 24 h. In HASMC cells treated with Avn C, the level of protein expression in MMP-9 was decreased with no variation in MMP-2 expression (Figure 3A). The mRNA level of MMP-9 was also reduced in HASMC cells with high concentrations of 0 to 100 μM Avn C (Figure 3B). Moreover, MMP-9 enzyme activity in TNF-α activated HASMC cells with Avn C treatment was significantly increased when compared to the TNF-α -treated control cells (Figure 3C). The results showed that, by treatment with Avn C, the mRNA and protein levels of MMP-9 enzyme were specifically decreased.

### Effects of Avn c on Secretion Levels of TNF-α-Activated Cytokines of IL-6, IL-1β and TNF-α in HASMC Cells

**FIGURE 4:**
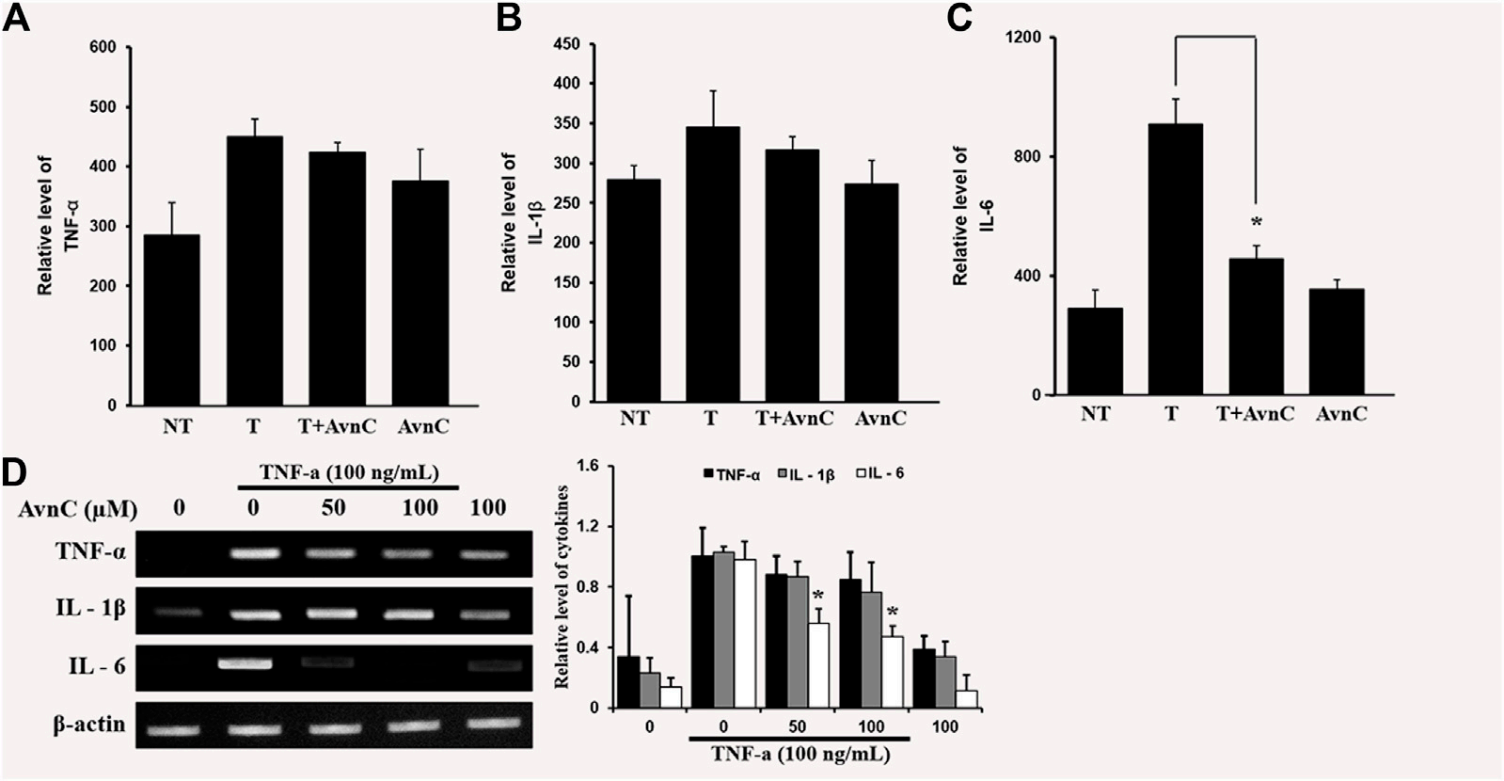
Effects of Avn C on TNF-α-induced cytokines secretion levels of IL-1β, IL-6 and TNF-α in HASMC cells **(A)** and **(B)** were not different in the secretion of IL-1 β and TNF- α cytokines with treatment by AvnC according to ELISA, but **(C)** Avn C attenuated TNF-α-induced IL-6 inflammatory cytokines. Results shown are representative of three independent experiments. Also, Avn C showed no effect on the total expression level of IL-1β and TNF-α, but it specifically decreased the expression level of IL-6 in TNF-α -stimulated HASMC cells by RT-PCR analysis **(D)**. Results are presented was mean ± SEM. *p < 0.05 indicates a significant difference from TNF-α-treated cells. NT, no treatment; T, TNF-α; T + AvnC, TNF-α + Avn C.

We assessed the effects of Avn C on secretions of well-known pro-inflammatory cytokines, such as IL-6, IL-1β, and TNF-α, by ELISA. In co-treatment of TNF-α and Avn C, there was no difference in the secretion levels of IL-1 β and TNF-α cytokines in the HASMC, but the secretion of IL-6 was inhibited (Figures 4A–C–C). Also, Avn C showed no effect on the total expression level of IL-1β and TNF-α, but it specifically decreased the expression level of IL-6 in TNF-α -stimulated HASMC cells by RT-PCR analysis (Figure 4D). The results showed that Avn C specifically inhibits IL-6 cytokine among the pro-inflammatory cytokines in HASMC cells.

### Effects of Avn C on TNF-α-Stimulated Nuclear Translocation of NF-kb in HASMC Cells

**FIGURE 5:**
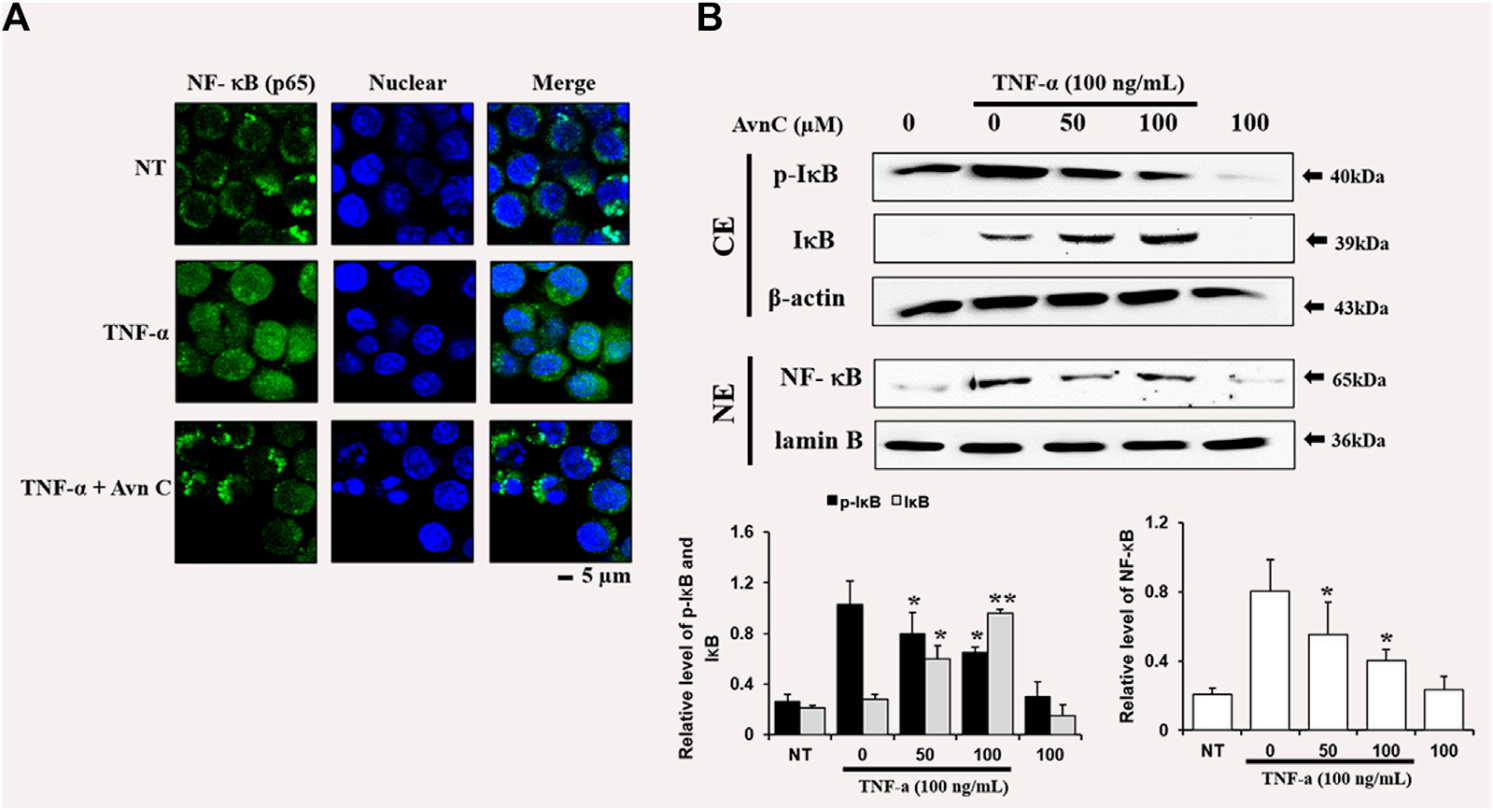
Effects of Avn C on TNF-α-induced nuclear translocation of NF-kB in HASMC cells **(A)** Transfer of NF-κB to the nucleus was detected by immunofluorescence assay. HASMC cells were immune-stained by FITC and Hoechst. Scale bars, 5 μm. NE, Nuclear extracts; CE, Cytosolic extract; NT, no treatment **(B)** Nuclear extracts were subjected to translocation to the nuclear region of the NF-κB subunit by Western blot. They are presented as mean ± SEM. *p < 0.05 and **p < 0.01, significant differences from the TNF-α treated cells. NT, no treatment.

To find the decrease in NF-κB translocation, we assessed the nuclear translocation level of the factor by direct immunofluorescence assay. The NF-κB translocation was decreased during processing of Avn C in the TNF-α activated HASMC, as detected by a fluorescence microscope, showing a specific reduction in NF- κB nuclear translocation (Figure 5A). Also, we investigated whether Avn C could control TNF-α-activated nuclear translocation of NF-κB in HASMC cells. Avn C inhibited the nuclear translocation from the cytosolic region to nucleus, because Avn C reduced cytosol nuclear translocation (Figure 5B). In this study, decreased IkB expressions and reduced the *p*-IkB level were observed, as expected (Figure 5B). These results show that Avn C could inhibit TNF-α-stimulated nuclear translocation of HASMC cells.

### Avn C Inhibits MAPK Phosphorylation in TNF-α-Activated HASMC Cells

**FIGURE 6:**
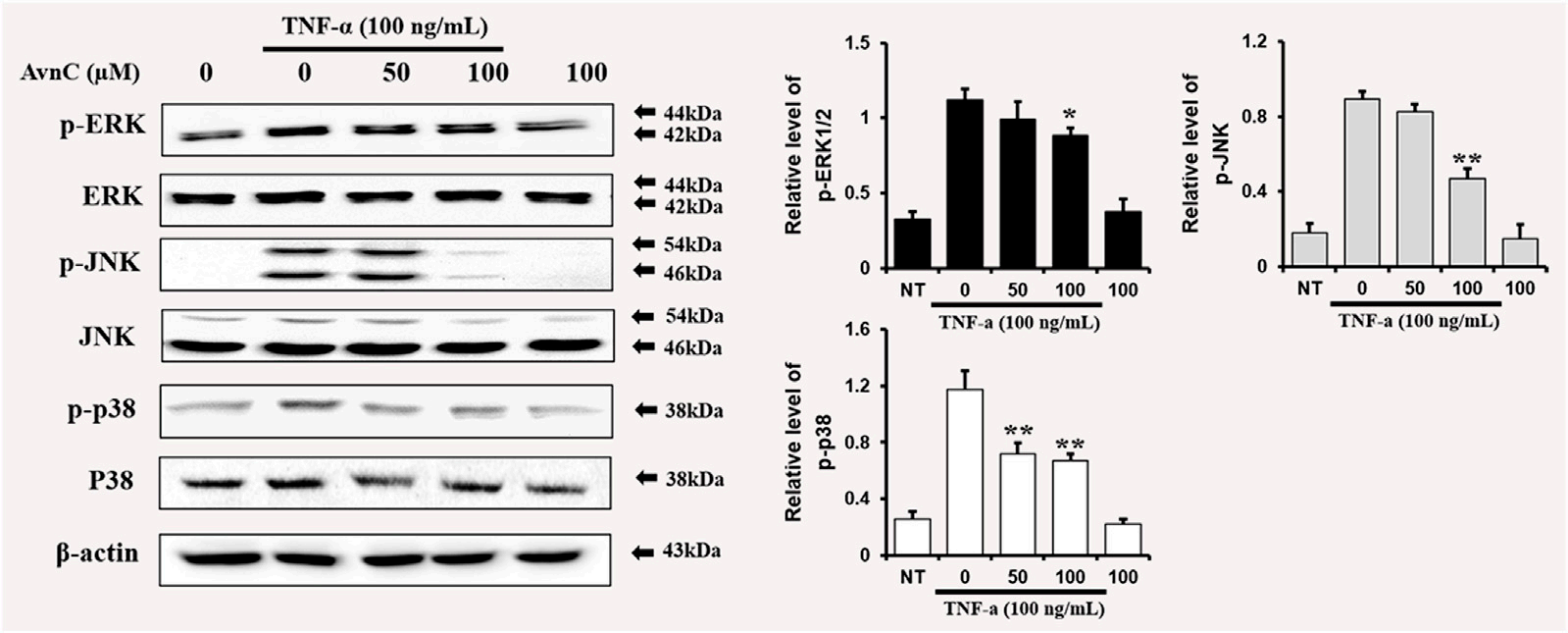
Avn C inhibits MAPK phosphorylation in TNF-α-activated HASMC cells.Levels of phosphorylated ERK, p38, and JNK were assessed by Western blotting. Levels of ERK, JNK, and p38 were indicated by loaded protein as individual control. Results shown are representative of three independent experiments. Results are presented as mean ± SEM. *p < 0.05 and **p < 0.01 indicate significant differences from TNF-α treated cells. NT, no treatment.

To find out whether Avn C affects MAPKs phosphorylation in TNF-α-induced HASMC cells, cells were treated with Avn C prior to TNF-α treatment, and then consequent levels of p38, JNK, and ERK phosphorylation were examined. Avn C decreased the phosphorylation levels of JNK, ERK, and p38 in TNF-α-activated HASMC cells (Figure 6). These results show that Avn C suppresses arteriosclerosis activity by downregulating the MAPK signaling pathway in HASMC cells induced by TNF-α.

## Discussion

Pathogenic atherosclerosis is associated with the secretion of MMP-9 and inflammatory cytokines to plasma and also in HASMC cells (Tayebjee et al., 2005; Nabata et al., 2008). Inflammatory cytokines, including IL-6, IL-1β, and TNF-α, are also directly associated with the MMP-9 expression in the *in vivo* and *in vitro* experimental subjects (Kang et al., 2005; Stanley and Lacy, 2010; Li et al., 2017).

**FIGURE 7:**
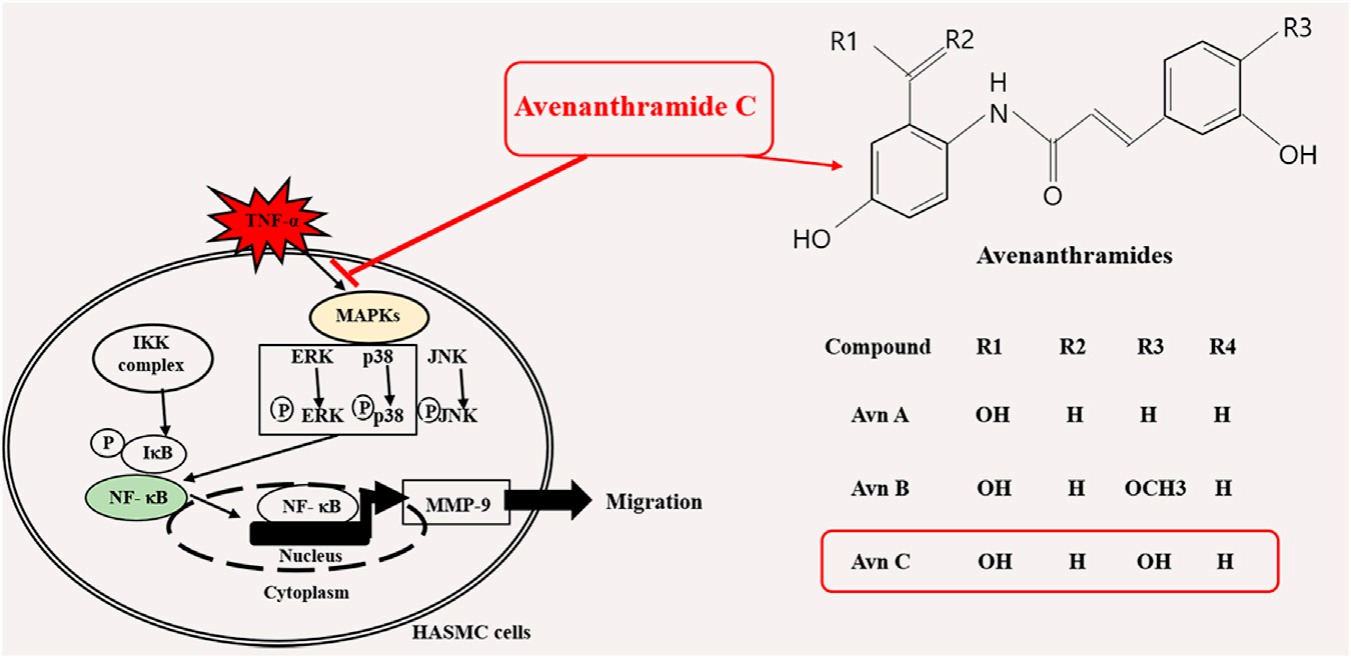
A schematic illustration of anti-atherosclerosis action of Avn C.

In this study, TNF-α-induced HASMC cells were used as an *in vitro* experiment model of arteriosclerosis (Kallenbach et al., 2003). We assessed the effect of Avn C (Figure 1) on TNF-α-activated atherosclerosis responses to understand the molecular mechanism underlying the anti-atherosclerotic potential of Avn-C. We confirmed the inhibition effect of Avn C on cell migration and wound healing in TNF-α-activated HASMC cells. These results indicate that the wound healing and migration activity of the vascular cells were suppressed by Avn C treatment (Figure 2). Avn C dose-dependently suppressed the increased MMP-9 protein and mRNA levels in HASMC cells upon TNF-α treatment. Avn C also inhibited MMP-9 enzyme activity (Figure 3). HASMC cells are known to produce inflammatory cytokines involving IL-6, IL-1β, and TNF-α during arteriosclerosis activity (Demyanets et al., 2011; Munoz-Garcia et al., 2006). Avn C specifically reduced IL-6 secretion in HASMC cells (Figure 4). Furthermore, we investigated whether Avn C could inhibit NF-κB nuclear protein translocation. Avn C suppressed nuclear protein translocation of NF-κB in TNF-α-stimulated HASMC cells dose-dependently (Figure 5). NF-κB is an important factor of the atherosclerotic manifest (Dabek et al., 2010). And the NF-κB regulator, IκB, has previously been identified as ensuring the level of nuclear translocation diminution (Ho et al., 2005; Ye et al., 2000). Next, we investigated whether Avn C influences the MAPKs phosphorylation. Avn C reduced the level of expression of ERK, JNK, and p38 phosphorylation in HASMC cells activated by TNF-α (Figure 6). The MAPK signaling pathway includes phosphorylation of p38, JNK, and ERK regulated by TNF-α (Chin et al., 1998; Zhao et al., 2019). Consequently, Avn C was shown to reduce the secretion of the cell migration and pro-cytokine through MAPK signaling, and to suppress arteriosclerosis progression (Figure 7). Therefore, we propose that Avn C could be a promising candidate for atherosclerosis diseases.

## Data Availability

The raw data supporting the conclusions of this article will be made available by the authors, without undue reservation, to any qualified researcher.

## Abbreviations

Avn C, avenanthramide C; COX-2, cyclooxyenase-2; DMEM, Dulbecco’s modified Eagle medium; DMSO, dimethyl sulfoxide; ECL, enhanced chemiluminescence; FBS, fetal bovine serum; FITC, fluorescein isothiocyanate; iNOS, inducible nitric oxide synthase; IL-1β, interleukin-1β; MAPKs, mitogen-activated protein kinases; MMP-9, matrix metallopeptidase 9; MTT, 3- (4,5-dimethylthiazol-2-yl)-2,5-diphenyl tetrazolium bromide; NF-κB, nuclear factor kappa-light-chain-enhancer of activated B cells; TBE, Tris-borate-EDTA; TNF-α, tumor necrosis factor-α
